# Development of mental health and psychosocial support (MHPSS) guidelines for deaf and hard of hearing children in the Gaza Strip

**DOI:** 10.1371/journal.pgph.0002427

**Published:** 2023-10-16

**Authors:** Nathaniel Scherer, Ramadan Hussein, Julian Eaton, Naim Kabaja, Ritsuko Kakuma, Tracey Smythe, Sarah Polack

**Affiliations:** 1 International Centre for Evidence in Disability, London School of Hygiene & Tropical Medicine, London, United Kingdom; 2 Atfaluna Society for Deaf Children, Gaza City, Palestinian Territories; 3 Centre for Global Mental Health, London School of Hygiene & Tropical Medicine, London, United Kingdom; 4 CBM Global Disability Inclusion, London, United Kingdom; 5 Division of Physiotherapy, Department of Health and Rehabilitation Sciences, Stellenbosch University, Stellenbosch, South Africa; The American University in Cairo, EGYPT

## Abstract

Deaf and hard of hearing children in the Gaza Strip may be at risk of mental health conditions and psychological distress, as a result of social exclusion and limited accessible communication. This article presents the process and research methods used to develop guidelines for schools in the Gaza Strip on mental health and psychosocial support for deaf and hard of hearing children. The process was guided by the GIN-McMaster guideline development checklist across four steps: (1) priority settings; (2) searching for evidence; (3) developing recommendations; (4) evaluation. Priority setting was spearheaded by local and international researchers, and a local steering committee comprised of deaf and hard of hearing representatives, school administration and staff, mental health specialists, family members and government officials. In searching for evidence, and in order to generate evidence-based recommendations for the guidelines, we utilised a scoping review of global mental health support for deaf and hard of hearing children and qualitative research with deaf and hard of hearing children and adults, families and teachers. Two pilot studies were conducted in mainstream and specialist educational settings as way of evaluation. The scoping review and qualitative research identified various content for the guidelines, including the importance of information on disability and deafness, promoting social inclusion and self-esteem, and accessible learning environments. The pilot studies demonstrated feasibility and acceptability among teachers and deaf and hard of hearing children, although teachers need sufficient support and resources to implement. Now finalised, the guidelines are being distributed to schools in the Gaza Strip to support the mental health and wellbeing of deaf and hard of hearing children.

## Introduction

The Gaza Strip has a population of 2.1 million, with 1.4 million being Palestinian refugees. The region has been affected by conflict and a deteriorating socioeconomic situation [1, 2]. The employment rate is 49% and access to clean water and electricity has been deemed a crisis by the United Nations [2]. These hardships are associated with mental health problems amongst youth, with evidence demonstrating high rates of depression, anxiety and post-traumatic stress disorder among children and adolescents [3–6]. Research has suggested that education and family support can improve the psychosocial wellbeing of youth in the Gaza Strip [3]. However, few young people receive formal mental health support, and many in the Gaza Strip experience challenges accessing mental health services [3, 7].

Data from the Palestine Population, Housing and Establishments Census 2017 estimate a disability prevalence of 5.8% in Palestine and 6.8% in the Gaza Strip [8]. The prevalence of disability among children aged 0–17 in the Gaza strip is 0.9%, with 0.2% reporting difficulty hearing. This equates to approximately 20,000 children in the Gaza Strip who experience difficulties with hearing. Evidence suggests that deaf and hard of hearing children worldwide may face difficulties in education, socialization, and community engagement [9–11], and are more likely than their hearing peers to report lower quality of life in school and social settings [11]. These negative experiences can impact emotional wellbeing and mental health, and deaf and hard of hearing individuals, both children and adults, have an increased risk of developing mental health conditions [12–20].

Deaf and hard of hearing youth in the Gaza Strip may face double-jeopardy with regards to maintaining psychosocial wellbeing; they may face stressors associated with living in a challenging environment, as well as those associated with being deaf or hard of hearing in a predominantly hearing world. Qualitative research in the Gaza Strip, conducted with deaf and hard of hearing children, parents of deaf and hard of hearing children, teachers of deaf and hard of hearing children, deaf and hard of hearing adults and mental health specialists, identified a number of stressors that put deaf and hard of hearing children in the Gaza strip at risk of mental health problems [21]. Stressors included lack of accessible communication, exclusion from community and community events, negative attitudes towards hearing impairment, and limited family knowledge on hearing impairment and deafness.

As deaf and hard of hearing children in the Gaza Strip are at risk of developing mental health conditions, there is need for structures to promote their wellbeing and to provide support if they are distressed. Mental health and psychosocial support (MHPSS) is a term that usually refers to promotion, protection and treatment for mental health and psychosocial wellbeing in emergency settings [22]. MHPSS takes a variety of forms, depending on context, resources and needs. Activities cover the full range of mental health needs, from (subclinical) distress, to treatment and prevention of mental health conditions, such as depression and anxiety. Activities include promotion of protective factors and behaviours that contribute to wellbeing, including social inclusion and access to community services [22]. Considering the important role that school plays in the lives of children and adolescents, educational settings are increasingly considered important environments in which to promote mental health and wellbeing. recognised schools as important environments in which to promote the mental health and psychosocial wellbeing of children [23]. Research with adolescents around the world identified school as a source of self-esteem, a setting for emotional support and an escape from adverse home environments, whilst also presenting academic pressures and a setting where children can experience abuse [24]. In a recent briefing note, the World Health Organization, UNESCO and UNICEF called on national governments to establish systems in schools that foster mental health and wellbeing, and effectively manage the mental health needs of students [25]. With deaf and hard of hearing children demonstrating unique psychosocial support needs, it is necessary to provide teachers and school staff in the Gaza Strip with tailored guidance on how to promote the mental health and psychosocial wellbeing of these children.

This article presents the methods and process used to develop guidance on MHPSS for schools to support deaf and hard of hearing children in the Gaza Strip, including information on evidence-generating activities to inform the content (such as a scoping review and qualitative research) and pilot testing of the guidelines to ensure contextual appropriateness. Throughout the study, we adopted participatory approaches to engage deaf and hard of hearing people and their families, to ensure that their voices and perspectives were heard, valued and included, in line with the principle espoused by the disability movement; “Nothing about us, without us” [26].

## Method

The MHPSS guidelines for deaf and hard of hearing children in the Gaza Strip were developed in line with the principles of the GIN-McMaster Guideline Development Checklist [27], developed by the Guidelines International Network (GIN) and McMaster University. The checklist comprises 18 key development steps and 146 individual items, including budgeting, stakeholder involvement and evaluation. We adapted the GIN-McMaster guideline development process to suit project objectives and resources. For ease of interpretation in this paper, we have summarised the development process under four key steps (numbers from the GIN-McMaster topic checklist are provided in brackets):

1. Priority setting (1–9)
2. Searching for evidence (10–12)
3. Developing recommendations (13–15)
4. Evaluation and use (16–18)

**Fig 1:**
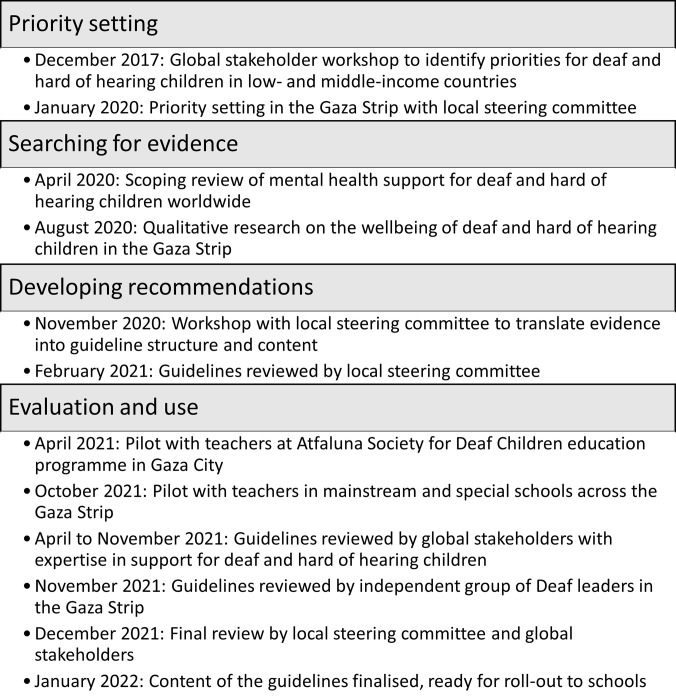
Guideline development process and timeline.

The project started during the early stages of the COVID-19 pandemic and this influenced the methods detailed and steps taken to develop the guidelines. Fig 1 provides an overview of the guideline development process.

### Priority setting (1–9)

Priority setting activities were conducted across the following steps:

#### 1. Global stakeholder workshop

In 2017, the International Centre for Evidence in Disability (ICED) at the London School of Hygiene & Tropical Medicine (LSHTM), CBM and DeafKidz International conducted a workshop to identify research and programmatic priorities to support deaf and hard of hearing children in low- and middle-income countries (LMICs). The group identified mental health and MHPSS as priorities for deaf and hard of hearing children. With little guidance available on MHPSS for deaf and hard of hearing children in LMICs, the group proposed developing guidelines that could be used by families, teachers, healthcare staff and other community stakeholders.

#### 2. Development programme in the Gaza Strip

In 2020, ICED established a partnership with CBM and Atfaluna Society for Deaf Children to develop guidelines on MHPSS for deaf and hard of hearing children in the Gaza Strip. This partnership brought together a research institute in global disability and mental health (ICED), an international disability NGO (CBM) and a local implementing partner focused on supporting deaf and hard of hearing children (Atfaluna Society for Deaf Children).

Guideline development was incorporated into a wider project being delivered by CBM and Atfaluna Society for Deaf Children, which aimed to provide training and interventions to promote the psychological wellbeing of children with disabilities, and in particular, deaf and hard of hearing children. The guideline development process was managed by ICED at LSHTM, in partnership with Atfaluna Society for Deaf Children. The team at ICED and LSHTM was comprised of researchers in disability and Global Mental Health, with experience in development guidance and manuals on a variety of health-related topics. The work at Atfaluna Society for Deaf Children was led by a Project Manager and Research Assistant. The Research Assistant was an audiologist at Atfaluna Society for Deaf Children, seconded to the research project for the duration. This collaborative research team comprise the authors on this paper.

#### 3. Local steering committee

To guide the development process and to set initial priority guideline topics, the team established a steering committee in the Gaza Strip. This group comprised: five deaf and hard of hearing people and representatives from organisations of deaf and hard of hearing people; two caregivers of deaf and hard of hearing children; four teachers of deaf and hard of hearing children; three mental health and psychosocial support specialists; and five representatives from government level institutions, including the Palestinian Ministry of Education and Higher Education. Group members were identified through the networks of Atfaluna Society for Deaf Children and the Palestinian Ministry of Education and Higher Education. Each member was provided a term of reference outlining their role as a steering committee member. The steering committee gathered with the research team three times throughout the development process, as described here and in subsequent steps. The meetings were held in-person, although the lead author from LSTHM attended virtually, with international travel restricted as a result of COVID-19. Typically, steering committee meetings were structured as half-day workshops, with opportunity for breakout sessions and group discussion. In the first meeting, the steering committee gave input to the research methodology, guideline development plan and priorities for the guidelines. The steering committee gathered two further times throughout the project, as described in the following sections.

#### 4. Consultation with global experts

In the early stages of the guideline development, the lead author consulted with international experts in mental health and support for deaf and hard of hearing children, including expertise in intervention development and delivery within low- and middle-income and humanitarian settings. Six experts from the UK, Syria, and Spain spoke to the lead author for approximately one hour via online communication technology, discussing priority topics, useful resources and other pertinent information. This information was used to guide the planning of the research components described.

### Search for evidence (10–12)

To inform the guideline content, we included two evidence-generating components: a systematic scoping review on mental health support for deaf and hard of hearing children globally; and qualitative research to explore experiences of deaf and hard of hearing children in the Gaza Strip and in schools, and their recommendations on MHPSS guideline content. Systematic review to search for evidence is explicitly recommended by the GIN-McMaster Checklist, although recommendations regarding qualitative research are absent. We conducted the additional qualitative research to provide an understanding of local contexts, cultural norms and attitudes to disability and mental health, and the experiences of deaf and hard of hearing children that may impact their wellbeing. There is a dearth of evidence on these areas, both in the Gaza Strip and globally, and this qualitative research was considered key to the development of contextually appropriate guidelines, as well as contributing to the wider evidence base.

#### 1. Scoping review

A scoping review was undertaken to map the global evidence on initiatives and interventions designed to protect and promote mental health and wellbeing support for deaf and hard of hearing children. Further details of the scoping review methods are available in the peer-reviewed article published in 2021 [28]. The review was guided by the framework developed by Arksey and O’Malley (2005) [29].

#### 2. Qualitative research in the Gaza Strip

To further understand the context and the experiences of deaf and hard of hearing children in the Gaza Strip, we conducted phenomenological qualitative research. This included in-depth interviews with 35 participants; 17 deaf and hard of hearing children aged 6–12 in mainstream and special schools, 10 parents or caregivers of deaf and hard of hearing children, and eight teachers of deaf and hard of hearing children from mainstream and special schools. Participants were purposively sampled from across the Gaza Strip (North, Gaza City, Central, South), and as following Patton’s maximum variation sampling [30], to reach a variety of ages, genders, severities of hearing impairment, location and types of school.

We also conducted three focus group discussions. The first comprised of representatives from Organisations of Persons with Disabilities (OPDs) and Deaf focused NGOs. Participants of this group included three deaf adults, one individual with a physical impairment and one with a visual impairment, who also works as a mental health counsellor. The second group included five school counsellors and two mental health professionals working with children in the community. The third group included seven special school teachers and six mainstream school teachers from across the Gaza Strip. These teachers were not the same as those included in the in-depth interviews.

Participant characteristics are presented in S2 Appendix. Data were collected in July 2020. The Research Assistant at Atfaluna Society for Deaf Children conducted the interviews and focus group discussions after receiving online training and remote supervision from ICED due to international COVID-19 travel restrictions. The interviews and focus groups themselves were conducted in-person, with safety measures adopted, as advised by the local government.

The interview guides were developed by the research team, piloted with six participants (two children, two caregivers, two teachers) and adapted as needed thereafter. Questions explored the experiences of deaf and hard of hearing children, families and teachers in education, health and community settings. For example, caregivers were asked, “How do people in the community view deaf and hard of hearing people?”, and children were asked, “How is the experience of learning in school?”. Specific questions were also asked on the planned MHPSS guidelines to elicit recommendations for the content and roll-out. For example, caregivers were explicitly asked, “What information should be included in the guidelines?”, and teachers were asked, “What support would you need to use the guidelines?”. Interviews were conducted orally in Arabic, with Palestinian sign language interpretation available. Children were interviewed individually, unless the child or caregiver desired otherwise. There was occasionally need for a family member to sit in as interpreter, when a child communicated through a signed language developed with family at home. Emotion cards were available to help children express their feelings. Each interview lasted 30–60 minutes. Five pilot interviews were conducted, in which to practice interviewing techniques and refine the topic guides. The data from these interviews has been included in the analysis. Audio-recordings of the interviews and focus group discussions were transcribed into English and thematically analysed [31]. Analysis was guided by the six steps outlined by Braun and Clarke (2006). First, the lead author familiarised themselves with the data, developing a codebook which was reviewed by co-authors. This codebook was piloted and iteratively adapted throughout the coding process. Coding was completed by the lead author in NVivo 12, with emerging themes identified, refined, and relationships compared between codes. The key themes were subsequently reviewed by co-authors and mapped against the data and emerging narrative. Finally, participant codes and narrative case studies were extracted. The themes were discussed with the steering committee to aid interpretation. Detailed analysis on factors contributing to the wellbeing of deaf and hard of hearing children in the Gaza Strip have been published elsewhere [21]. In this paper, we present findings relevant specifically to guideline development.

### Developing recommendations (13–15)

Next, we held a series of participatory workshops with the steering committee, in order to develop consensus on the structure and content of the guidelines. During the workshops, key themes and recommendations emerging from the evidence-generating activities were presented. These were used by the group to collectively agree on the planned structure and content of the guidelines.

Two workshops were held with steering committee members at this stage. The first was held with parents, deaf adults, teachers, head teachers and mental health specialists. The second was held with the representatives from the Palestinian Ministry of Education and Higher Education and UNRWA. This separation aimed to reduce power dynamics and to promote a bottom-up approach to guideline development. The first session developed the core outline of the guidelines, with the second session focused more on review, comment and consideration of the recommendations in the context of education systems and infrastructure in the Gaza Strip. From these recommendations, the lead author developed the initial draft of the guidelines for pilot.

The steering committee gathered for a third time once the first draft of the guidelines was complete, in order to review the draft and discuss amendments to the content. The first draft was also reviewed in an in-person workshop with a separate group of Deaf leaders in the Gaza Strip, who provided their opinions on the content and needed changes. Further, global stakeholders with expertise in supporting deaf and hard of hearing children reviewed the first draft via email, including Al Damair Society, Dair Al Balah Society for Rehabilitation, and Al Amal Society for Rehabilitation (all located in the Palestinian Territories), and the World Federation of the Deaf.

### Evaluation and use (16–18)

Once redrafted, the guidelines were pilot tested twice in the Gaza Strip. Initially, the plan was to test the guidelines with teachers in mainstream and special schools, but with widespread school closures as a result of COVID-19, the team had to find other ways to test the guidelines. Atfaluna Society for Deaf Children had dispensation to continue running a face-to-face education programme throughout the pandemic and we decided this would serve as the first pilot site. Once mainstream and special schools re-opened, a second pilot was held.

#### 1. Pilot one: Atfaluna Society for Deaf Children education programme (April 2021)

The education programme at Atfaluna Society for Deaf Children provides educational opportunities, as well as broader support and counselling for children with disabilities and education challenges. All 10 teachers involved in the programme agreed to take part in the first pilot of the guidelines. Each received a half-day orientation on the guidelines and then used the guidelines within their class for three-weeks.

A pre- and post-questionnaire, based on Bowen’s feasibility framework, was administered to teachers before and after guideline use [32]. The questionnaire explored four of the eight components of the framework: Acceptability (To what extent the guideline is judged as suitable?), Demand (To what extent the guideline is likely to be used?), Implementation (To what extent the guideline can be successfully delivered by teachers?) and Practicality (To what extent can the guideline be implemented with existing means and resources?). Additionally, we asked questions on knowledge about disability, deafness and mental health support among teachers before and after using the guidelines. We further included open-ended questions, in which participants could make suggestions on amendments to the content and the format.

After completing the pilot and questionnaire, the 10 teachers participated in a half-day workshop with the research team, to discuss their experience of using the guidelines and suggestions for improvement.

#### 2. Pilot two: Mainstream and special schools in the Gaza Strip (August 2021)

Once schools re-opened, we proceeded with the second pilot, within mainstream and special schools. In total, 30 teachers were included in the second pilot across nine schools; 19 from mainstream schools, 11 from special schools. Teachers were purposively selected to represent a diverse group across gender, age group taught, region and school type (mainstream/special).

As with the first pilot, teachers received orientation, used the guidelines with their class (for four-weeks) and completed pre-and post- questionnaires They also participated in a half-day workshop to discuss their experience and suggestions for improvement. Additionally, we spoke to the 10 head teachers of the included schools, with regards to their views of the teacher’s experience and feedback.

It is important to note that pilot two was slightly disrupted by COVID-19, with some schools closing for periods of the pilot and school days shorter than usual. No school was closed for the duration of the pilot, however.

Based on the feedback in pilots one and two, the guidelines and orientation were updated, as described in the results. Members of the steering committee and the World Federation of the Deaf were invited to provide final review and comment to this updated draft via email.

### Ethical approval

Ethical approval was provided for the qualitative research and pilot studies by the ethics board at the London School of Hygiene & Tropical Medicine (Ref: 19144 and 25113, respectively) and the Palestinian Health Research Council (Ref: PHRC/HC/697/20 and PHRC/HC/855/21). Informed written consent was obtained from all participants. Caregivers and guardians provided informed consent for children. Assent was sought from children using a simplified information sheet. For clarification, caregiver consent and child assent was obtained because of the children’s age, not because the children were deaf or hard of hearing.

## Results

### Priority setting (1–9)

From the outset, the project partners identified schools as a setting to provide the MHPSS guidelines, given the benefits of early intervention, the important and regular contact between teachers and deaf and hard of hearing children, and the importance of the setting to aspects of importance to psychological wellbeing, such as social inclusion, peer-support and education [33–35].

In the early stages of the project, both the local steering committee and international experts recommended that the guidelines should focus on improving teachers’ understanding of hearing impairment and deafness in the Gaza Strip. They believed that before supporting deaf and hard of hearing children’s mental health and wellbeing, teachers must first have insight into what deaf and hard of hearing children experience. This included learning about child development, communication and learning preferences of deaf and hard of hearing children. When identifying priority topics for MHPSS specifically, we were told that teachers in the Gaza Strip needed information on identifying mental distress and information on support to provide. This should also include promotion and prevention techniques to promote wellbeing in the classroom. The committee said that it was vital to include contextually appropriate MHPSS activities that teachers would feasibly be able to deliver with the resources available. They recommended searching for evidence on low-intensity interventions, such as safe spaces for deaf and hard of hearing children, emotional literacy training and peer-support.

### Search for evidence (10–12)

#### 1. Scoping review

Results of the scoping review of MHPSS interventions for deaf and hard of hearing children are published elsewhere [28]. In summary, 27 articles were included, the majority (81%) of which were sourced from high-income settings. Interventions were typically therapy-based (30%) or skills training (30%) and included ice-skating, parent-child interaction therapy and resilience training.

From the review, key lessons taken to inform content of the MHPSS guidelines included the importance of: peer-support building resilience, emotional literacy and behavioural management, physical activity, knowledge and awareness of disability and hearing impairment, appropriate communication, accessible physical environments and materials, collaboration between teachers, parents and children, and conducting pilot studies (Table 1).

**Table 1:** Summary of content derived from evidence-generating activities.

Evidence generating activity	Content included in the guidelines
Scoping review	• Importance of peer-support • Promoting emotional literacy • Behavioural management • Supporting physical activity • Improving knowledge and awareness of deafness and hearing impairment • Communication is key • Developing accessible environments • Importance of collaboration
Qualitative research	• Information on deafness and hearing impairment, improve knowledge of teachers, parents and other children • Promote social inclusion, include a buddy system with a hearing partner • Build self-esteem, give deaf and hard of hearing children responsibility in the classroom • Improve emotional literacy in deaf and hard of hearing children • Collaborate with school counsellors to help children needing support • Create an accessible learning environment, consider appropriate communication and classroom setup • Develop partnership between parents and teachers to provide continuity of support

#### 2. Qualitative research

The qualitative research uncovered key themes that contribute to psychological distress and psychological wellbeing of deaf and hard of hearing children in the Gaza Strip, including language deprivation, community attitudes and family support. Findings relevant to these themes have been published elsewhere [21]. Recommendations for the guidelines extracted from these data included the need for appropriate communication, sign language training, promoting social inclusion of deaf and hard of hearing children in school, greater provision of assistive technology and training, community awareness and reduction of stigma, and parent support and sensitisation.

Key themes derived from data specific to the MHPSS guidelines included the desire from teachers for resources and guidance, the need for information on disability and deafness, actions to promote social inclusion, developing children’s self-esteem and emotional literacy, how to build inclusive learning environments, and how teachers and parents can work together.

With regard to perceptions of the proposed MHPSS guidelines and their recommendations for content, the majority of participants reacted positively. Many teachers, especially those in mainstream schools, stated that they needed and wanted training and guidance on supporting deaf and hard of hearing children.

“[*When asked if the guide will be useful] Of course it will be*. *It is going to include new information and knowledge that a teacher can realize and apply with the children*. *As someone still learning*, *this will enhance my knowledge*, *because you still are unaware of many things*. *The guide will help in filling the gaps*, *especially in solving problems*.*” (Teacher at a special school)*

Key content areas that participants felt should be in the guidelines included information on disability, hearing impairment, deafness, Deaf culture and mental health (Table 1). We were told that many teachers, school staff and hearing children didn’t know much about these topics and this sensitisation was vital if deaf and hard of hearing children were to be included and respected in the classroom.

Exclusion and isolation were said to be common for deaf and hard of hearing children, especially in mainstream schools, and this exclusion was an important determinant of mental distress. Suggestions to improve inclusion included encouraging group work and extra-curricular activities, to build relationships and friendships. Some participants also recommended a buddy system, in which a hearing child is partnered with a deaf or hard of hearing child in a mainstream school to help them learn and feel included in the classroom. Many deaf and hard of hearing children expressed an interest in participating in social groups with other deaf and hard of hearing children, citing a common experience and understanding.

In addition to understanding hearing impairment and deafness, participants also requested information on assistive technology, particularly hearing aids and cochlear implants. This included practical information on basic maintenance, such as changing hearing aid batteries. Many teachers expressed that they and hearing children in mainstream schools did not understand what the assistive technology was nor its purpose. deaf and hard of hearing children told us of examples in which other children removed their hearing aid, thinking they were listening to music.

*“Once the teacher was afraid of her hearing aid*, *because it was making a buzzing sound*, *which is something annoying*. *When the teacher saw the hearing aid she was frightened*. *[*…*] They should be educated about hearing aids and such problems*.*” (Father of a deaf child aged 10–12)*

To protect against mental distress, participants said that teachers must help develop a child’s sense of identity and self-esteem. Teachers were encouraged to give deaf and hard of hearing children responsibility in the classroom, such as being class secretary for a period. To further support mental health and wellbeing, participants wanted information on emotional literacy and how to help children understand their own feelings and emotions. It was also vital that teachers be aware of how to recognise mental distress and the process to follow if they identify mental distress, including referral to school counsellors. School counsellors urged teachers to work with them when they have a concern, although teachers in the study told us that this collaboration was often not strong.

“*Interviewer*: *What do you think would contribute to improving the psychosocial status of deaf and hard of hearing children*?

*Respondent*: *By improving their skills*. *For example*, *look at what the child loves and work on improving this and improving the child’s ability in this field*. *When a child with a disability is left behind because of their disability*, *this impacts their mental health and causes them psychosocial problems*. *We need to improve their skills*, *look into their talents and work on developing them*. *This would improve their self-trust and help them overcome mental health problems*.” *(Counsellor at a mainstream school)*

As well as an explicit focus on mental health and wellbeing, participants also suggested that the guidelines include information on how to provide an accessible and inclusive learning environment, especially in mainstream education. This was echoed by the steering committee in early-stage meetings. Recommended areas to focus on included communication, accessible teaching materials and the physical environment, including consideration of seating arrangements and minimising distracting noises.

Finally, throughout the qualitative research, participants emphasised the need for teachers and parents to work together. Many parents wanted to be involved and to help teachers support their child’s learning and wellbeing. They also wanted to be given the tools to continue support at home. Regular communication was said to be needed and some participants suggested a joint notebook, in which teachers and parents could keep a track of a child’s experience at school and home, and actions being taken.

*“We always prefer parents to be aware of persons with disabilities needs*, *sign language and support services available to them*. *If I’m the only one who communicates with the child and there’s no communication at home*, *our efforts will be in vain*. *This is why we keep encouraging parents to communicate in sign language with their deaf and hard of hearing children*. *It’s really dangerous to depend on the teacher only*.” *(Teacher at a special school)*

One father told us of an instance in which his hard of hearing son, attending a mainstream school, was exhibiting challenging behaviours and difficulties with concentration and learning. The father worked with the teacher and discussed how much of this arose from inequalities and exclusion in the classroom. The teacher was responsive and made efforts in the classroom to promote the inclusion of the hard of hearing child. The child’s behaviours improved thereafter.

In addition to guideline content, participants also discussed the eventual roll-out of the guidelines across mainstream and special schools in the Gaza Strip. Teachers expressed a need for institutional awareness and buy-in to support them in implementing the guidance in their classroom. It was said to be particularly important that school administration, head teachers and staff understand the value of the guidelines and give teachers the support they need. Teachers also said they would need training on using the guidelines, including ongoing mentorship from school counsellors.

### Developing recommendations (13–15)

Table 2 presents the guideline structure and content summary developed with review by steering committee, Deaf leaders and experts in mental health and deaf and hard of hearing children. Throughout the guidelines, teachers are provided with practical activities to support deaf and hard of hearing children. Examples include role-play to help demonstrate positive behaviours and setting up a ‘buddy’ system, pairing a deaf or hard of hearing child with a hearing peer, who can help them navigate school life.

**Table 2:** Summary of guideline structure and content.

Section	Summary of content
Understanding mental health and psychosocial support	• What is mental health and wellbeing? • Risks to mental health and wellbeing • Mental health in the Gaza Strip
Understanding disability	• What is disability? • Barriers experienced by people with disabilities • Impact of disability • Disability rights
Understanding hearing impairment and deafness	• What is hearing impairment? • Severity of hearing impairment • What is deafness? • Deaf culture • Assistive technology (e.g. hearing aids) • Myths and facts
Impact of hearing impairment and deafness	• Communication and language • Influence on social skills • Impact on education • Impact on mental health
Supporting deaf and hard of hearing children	• Promoting understanding and information on hearing impairment and deafness • Addressing stigma • Promoting social inclusion • Accessible communication • Accessible classroom environment • Accessible learning materials
Supporting the mental health and wellbeing of deaf and hard of hearing children	• Building positive deaf and hard of hearing identity • Developing inclusion and friendship • Promoting self-esteem • Emotional literacy • Resilience training • Promoting positive behaviour • Physical health
Identifying a mental health issue	• Signs of mental distress • Steps to take on identification, including referral to school counsellor
Collaboration	• How to coordinate with other teachers • How to collaborate with school counsellors • Working in partnership with parents and caregivers • Continuing support at home
Appendices	• List of hearing services in the Gaza Strip • List of MHPSS services in the Gaza Strip • Simple Palestinian sign language • Hearing aid care • Creating an inclusive environment: self-assessment for teachers • Questions to ask deaf and hard of hearing children about their school experience • Emotions cards

### Evaluation and use (16–18)

The results of pilots one and two have been summarised against the four areas of feasibility from Bowen’s framework [32]. Data from pilot one and two have been combined. Responses from open-ended questions are provided.

#### Acceptability: To what extent were the guidelines judged as suitable, satisfying or attractive to teachers?.

Across the two pilots (n = 40), 90% of teachers reported that the guidelines met/exceeded their expectations, and 95% were satisfied/very satisfied with the guidelines. Teachers reported the perceived value of the guidelines, that were seen to be rich in detail and easy to use. The simple language was easy to understand and commonly cited a positive of the guidelines

*“I am satisfied with the content*. *It contains information that has added to my knowledge and made me aware of some of the problems facing deaf and hard of hearing children in my classroom*.” *(Teacher at mainstream school)*

With regards to perceived effect of the guidelines, most (81%) of the teachers reported the guidelines to moderately/significantly improve their knowledge of MHPSS, with 76% reporting moderately/significantly improved knowledge on MHPSS for deaf and hard of hearing children specifically. There was reported increased understanding of the challenges facing deaf and hard of hearing children and the need for MHPSS, increased understanding of MHPSS and how to support wellbeing, and increased understanding of inclusion within the classroom. Among those that reported a slight improvement in knowledge only, many stated that they needed more time to become familiar with the topics of the guidelines.

Nearly all (90%) teachers told us that they had moderately/significantly changed their actions and behaviours in their classroom. Teachers told us that they have started to speak to deaf and hard of hearing children and families about their needs, including communication. Teachers promoted inclusion in the classroom and held sessions in mainstream schools to explain hearing impairment, deafness and hearing aids to other students. Many encouraged deaf and hard of hearing children to talk to their class on their hearing impairment and on their use of hearing aids. Teachers also reported actively encouraging deaf and hard of hearing children to participate in classroom activities.

Further, 79% of teachers reported that deaf and hard of hearing children in their classrooms moderately/significantly accepted use of the guidelines and many teachers reported positive outcomes among deaf and hard of hearing children. Deaf and hard of hearing children and their families had reacted well to teachers showing an interest in their needs, and the children noticed a difference in the teacher’s and classmates’ understanding and approach with them. According to teachers, deaf and hard of hearing children were included more by their peers and they were more active in class. Many reported that deaf and hard of hearing children’s confidence had grown and they were more open with the class and teachers. Teachers reported improved relationships between them and the children.

*“We are in a better relationship now*. *They love the activities we are doing together and they ask for more*.” *(Teacher at education programme)*

Teachers at special schools recognised that much of the information in the guidelines was not relevant to them, as they already have a strong understanding of disability and hearing impairment, but the elements of mental health were important and they perceived a benefit to distribution across special schools.

#### Demand: To what extent were the guidelines likely to be used by teachers?

All teachers said that they would be likely/very likely to use the guidelines in the future. Overall, 83% of teachers believed that the guidelines would be moderately/significantly useful for teachers to support the mental health and wellbeing of deaf and hard of hearing children. The remaining reported it slightly useful, reporting reservations over capacity.

When asked how often they apply the recommendations of the guidelines, 36% of teachers reported weekly, 48% two to three times per week, and 17% daily. Teachers told us that they often referred to the guidelines to understand a child’s behaviour and to identify concerns.

*“I read and referred to the guidance frequently*. *I wanted to be more aware of all aspects of MHPSS and to notice any concerns with the children I am teaching*.” *(Teacher at mainstream school)*

The majority (93%) of the teachers said that they were moderately/very confident to use the guidelines in the future, with all teachers reporting that the induction on the guidelines was needed and useful.

#### Implementation and practicality: To what extent could the guidelines be successfully delivered to deaf and hard of hearing children in mainstream and special schools using existing resources?

Although teachers were interested to use the guidelines, results from the questionnaire and workshop highlighted concerns among teachers about their capacity to use them; 40% said the guidelines would be difficult/very difficult to use alongside their current teaching schedule and activities. This increased to 53% for mainstream and special school teachers in pilot two only. For some, this reflected the situation related to COVID-19 and shorter school days. Some believed they would have capacity once normal schedules resumed. Others said it was difficult to find the time to read and implement the guidelines, although there was acknowledgement that the pilot had run for a relatively short-time only and with more time they would be able to better take in the information and make changes.

The majority of teachers said that they needed practical support from the school administration to implement the guidelines, including provision of accessible learning materials and resources, time in their schedule, and ongoing capacity-development.

*“Teachers need training and support from the school administration to be able to apply the guideline*… *If this would be possible*, *the guideline will be very helpful to ensure needed positive change*.” *(Teacher at education programme)*

Some of the teachers reported that they had received ongoing advice and support from school counsellors as they learned to use the guidelines during the pilot, citing this a significant facilitator to guideline implementation. Nearly all (95%) said that the orientation was needed to prepare teachers to use the guidelines, an important consideration when aiming to scale-up guideline use across all schools.

Concerns of teacher capacity were further reported by head teachers. In 2021, there had been a shift in the school system. The new structure consists of four quarters (instead of two semesters); the children go to school for two months, then have exams, followed by 10 days off. This is repeated four times throughout the year. We were told this results in shorter and busier days and teachers have more work to do. The teachers felt this change fostered a greater focus on academic achievement and less time for wider activities, such as mental health and wellbeing support. This was reported important to consider when rolling-out the guidelines across the Gaza Strip.

#### Adaptations to the guidelines

After completing pilots one and two, small adaptations were made to the guidelines, based on feedback from the teachers. Amendments included additional information on the impact of hearing impairment of child development, more examples on how to promote social inclusion of deaf and hard of hearing children in classrooms, and clarity on the referral process when a teacher identifies a child in distress. Additionally, teachers asked for more information on the impact on parents of deaf and hard of hearing children and more information on how to involve parents in their child’s education. After making these amendments, the content was reviewed online by steering committee members and the World Federation of the Deaf. The guidelines were subsequently finalised, ready for use in schools in the Gaza Strip.

#### Guideline use

With the guidelines complete, Atfaluna Society for Deaf Children, in collaboration with the Palestinian Ministry of Education and Higher Education, are systematically rolling-out the guidelines across mainstream and special schools in the Gaza Strip. To aid this roll-out, they have developed a dissemination plan, including presentation events at schools. They have also developed a training programme for parents and school staff, including teachers, administration and counsellors, on mental health and psychosocial support, including use of these guidelines.

The guidelines are now available online in English and Arabic, and we have encouraged others to adapt and use the guidelines as suitable.

## Discussion

This article has described the participatory process of developing guidelines on MHPSS for deaf and hard of hearing children in the Gaza Strip, including detail on activities to generate evidence-based content and evaluation to assess feasibility and acceptability. Developing the guidelines throughout the COVID-19 pandemic resulted in challenges, but using the GIN-McMaster checklist provided best-practice guidance to support the development process.

### Practicality of the guidelines

From the outset, the research team and local steering committee identified schools as the priority setting for these MHPSS guidelines, congruent with long-standing research and practice on the suitability of schools as a natural setting to deliver mental health support [36, 37]. Children spend a substantial amount of their time in these settings and schools play a significant role in a child’s social and emotional development, providing opportunity for peer-interaction, behavioural management, and academic progression [37]. Schools often have existing resources and support systems on which to scaffold mental health support, whether that be prevention, promotion or low-intensity intervention, and they are well-placed to connect children and families to further services. In the Gaza Strip, schools are meant to have a counsellor on staff, who can offer direct support or refer to external mental health services. However, our findings aligned with other research highlighting that teachers often have limited mental health literacy, skills and confidence with which to identify mental health needs and provide support [38–40]. Lack of training is a key barrier to teacher-delivered mental health support in different settings [39]. Teachers need training and guidance if they are to support the psychosocial wellbeing of children in schools, supporting the rationale for the guidelines developed.

The pilot phase was a key step in assessing the feasibility and acceptability of the guidelines. Positive feedback suggested that the guidelines included useful information, which was readily actioned by many of the teachers. However, it is important to recognise findings regarding teacher capacity and availability of time. Over half of the teachers in pilot two and 40% overall reported that it was difficult to use the guidelines alongside their current teaching duties. This is an issue seen with many school-based mental health initiatives; teachers’ experience a conflict in their role, stretching their capacity [39, 41]. This is a crucial consideration when looking at the long-term viability and scalability of these guidelines in the Gaza Strip and other such mental health support structures in schools. Support of school leadership, both national and local, is needed to facilitate teachers in the delivery of mental health support [41]. Leadership must provide appropriate time and resources to teachers, to ensure that they are not overburdened. This includes hiring enough school counsellors to provide support to teachers aiming to promote wellbeing in their classrooms, as well providing more direct support to children who experience mental distress. In this development process, we included the Palestinian Ministry of Education and Higher Education from the outset, to encourage buy-in and input to the content and scale-up of the guidelines. Their support to schools and teachers is vital to the viability of guideline use. Further, the results of the pilot highlight the value of the half-day orientation and ongoing mentorship from school counsellors to support teachers to implement the guidelines. Building in these systems is important when scaling-up to all schools. As mentioned, Atfaluna Society for Deaf Children are training school staff, including school counsellors, on psychosocial support and use of the guidelines, in order to promote sustainable and effective utilisation of the guidelines.

With the guidelines being rolled-out to schools, it is important that national actors recognise that many deaf and hard of hearing children in the Gaza Strip may not be attending school and will not benefit from the support provided in these guidelines. Estimates from the Population, Housing and Establishments Census 2017 suggest that 35% of adolescents with disabilities aged 16+ have never enrolled in school [8]. It is imperative, therefore, that additional strategies are developed to support the mental health and wellbeing of deaf and hard of hearing children without access to educational settings.

### Challenges and mitigations in the guideline development process

The data collection and development process were impacted by the COVID-19 pandemic as well as periods of heightened conflict in the Gaza Strip, namely May 2021. In the context of lockdown measures, online data collection was considered for the qualitative research. However, we believed this would reduce rapport and therefore data quality. Further, this may have been challenging for children and some deaf and hard of hearing participants using a sign language interpreter. When lockdown measures were lifted, we interviewed participants in-person, following local safety guidance. With international travel limited, the UK researchers could not travel to the Gaza Strip and therefore they supported remotely, training the local researcher and attending interviews over the internet (using Zoom), utilising ICED qualitative remote data collection tools to structure the process [42].

The initial planned pilot was also disrupted, following increases in COVID-19 cases and lockdown measures in 2021. However, as a result we conducted two pilots to understand the feasibility and acceptability of the guidelines. School closures also highlighted the need for us to include information on how teachers can continue to provide MHPSS remotely, in case of future lockdowns. This is particularly relevant in a context of regular crises impacting on the delivery of education.

### Strengths of this guideline development process

As recommended in the GIN-McMaster checklist, we formed a multi-disciplinary steering committee, with expertise and knowledge on supporting the inclusion and wellbeing of deaf and hard of hearing children in the Gaza Strip. This steering committee was instrumental in guiding the development and content of the guidelines, providing both input and critical review. Utilising their knowledge throughout the process encouraged discussion and consensus on the tools that would best equip teachers to support deaf and hard of hearing children in their classrooms. Utilising participatory approaches in the workshops was vital to their success [26].

The appropriateness of the content to the needs of deaf and hard of hearing children was achieved by generating evidence on best practice. Conducting qualitative research enhanced our understanding of the context for deaf and hard of hearing children in the Gaza Strip, enabling tailored initiatives in the final guidelines. This qualitative research further added to the literature on mental health and wellbeing for deaf and hard of hearing populations in low- and middle-income countries, an area lacking in evidence.

The evaluation of the guidelines further strengthened the appropriateness of the guidelines for use by teachers in the Gaza Strip and resulted in a real-world understanding of its use in practice and what teachers could reasonably achieve in their classrooms. Having the guidelines peer-reviewed by leading deaf and hard of hearing global stakeholders added further validity to the guidelines and has created an opportunity for these guidelines to be adapted to other contexts and settings, which we encourage.

### Limitations of the guideline development process

The sample size of the pilot was small and it would have been valuable to conduct a larger scale pilot to understand feasibility and acceptability. Further, each pilot could only run for three to four weeks (as a result of COVID-19 restrictions and heightened conflict in the region). Teachers reported needing more time to utilise the guidelines and we do not have an understanding about sustained use of the guidelines.

We also did not have the capacity or time to conduct in-depth qualitative assessment during the pilot and were restricted to feedback through questionnaires and the workshops. Although these included open-ended questions, the understanding of the guidelines in practice would have been strengthened by further in-depth qualitative assessment with teachers. Additional qualitative research with deaf and hard of hearing children about their experience of guideline implementation would have further strengthened our understanding.

### Future of the guidelines

The MHPSS guidelines are being rolled-out across mainstream and special schools in the Gaza Strip, in partnership with the Palestinian Ministry of Education and Higher Education. These guidelines are freely available online in English and Arabic, and we encourage governments and service providers in similar settings to adapt the guidelines to their context.

This study and development process identified a need for information and guidance on inclusive learning environments, communication, assistive technology, social inclusion, emotional literacy, and partnership with parents. These findings are consistent with previous literature from different settings, highlighting common needs in the context of MHPSS for deaf and hard of hearing children [35, 43–47]. However, it is important to respond to these needs in a context-specific manner, and adapting the guidelines developed in this study should include appropriate research methodologies to ensure contextual relevance. When adapting to other settings, it is important to do so in partnership with deaf and hard of hearing groups, parents, teachers, and other key stakeholders.

### Future research

We recommend conducting a longer-term assessment of guideline feasibility and acceptability. Assessing deaf and hard of hearing child outcomes, including social inclusion and mental health status, would also be beneficial. In addition, it would be useful to conduct a process evaluation on the guideline roll-out procedures and long-term implementation.

## Conclusion

This article has described the process of developing MHPSS guidelines for deaf and hard of hearing children in the Gaza Strip. This process was supported by a structured guideline development framework, and adoption of participatory approaches, evidence-generating activities and evaluation. The guidelines were found to be acceptable and feasible to implement, with support from school administration. These guidelines will aim to support to psychosocial wellbeing of deaf and hard of hearing children in the region and we encourage adaptation of these to other contexts.

## Supporting information

S1 Appendix Questionnaire on inclusivity in global health. (DOCX)

S2 Appendix Qualitative participant characteristics. (DOCX)
